# Diabetes Impairs the Vascular Recruitment of Normal Stem Cells by Oxidant Damage, Reversed by Increases in pAMPK, Heme Oxygenase-1, and Adiponectin

**DOI:** 10.1634/stemcells.2008-0800

**Published:** 2009-02

**Authors:** Gianmario Sambuceti, Silvia Morbelli, Luca Vanella, Claudia Kusmic, Cecilia Marini, Michela Massollo, Carla Augeri, Mirko Corselli, Chiara Ghersi, Barbara Chiavarina, Luigi F Rodella, Antonio L'Abbate, George Drummond, Nader G Abraham, Francesco Frassoni

**Affiliations:** a Department of Internal Medicine, Nuclear Medicine, University of Genoa, S. Martino HospitalGenoa, Italy; b Advanced Biotechnology CenterGenoa, Italy; c Departments of Medicine and Pharmacology, New York Medical CollegeValhalla, New York, USA; d CNR Institute of Bioimages and Molecular PhysiologyMilan, Genoa Section, Italy; e Centro Cellule Staminali e Terapia Cellulare, S. Martino HospitalGenoa, Italy; f Scuola Superiore Sant'AnnaPisa, Italy; g Department of Biomedical Science, University of BresciaBrescia, Italy

**Keywords:** Endothelial progenitor cells, NO, pAMPK, HO-1, Vascular repair, CD31

## Abstract

**Background:**

Atherosclerosis progression is accelerated in diabetes mellitus (DM) by either direct endothelial damage or reduced availability and function of endothelial progenitor cells (EPCs). Both alterations are related to increased oxidant damage.

**Aim:**

We examined if DM specifically impairs vascular signaling, thereby reducing the recruitment of normal EPCs, and if increases in antioxidant levels by induction of heme oxygenase-1 (HO-1) can reverse this condition.

**Methods:**

Control and diabetic rats were treated with the HO-1 inducer cobalt protoporphyrin (CoPP) once a week for 3 weeks. Eight weeks after the development of diabetes, EPCs harvested from the aorta of syngenic inbred normal rats and labeled with technetium-99m-exametazime were infused via the femoral vein to estimate their blood clearance and aortic recruitment. Circulating endothelial cells (CECs) and the aortic expression of thrombomodulin (TM), CD31, and endothelial nitric oxide synthase (eNOS) were used to measure endothelial damage.

**Results:**

DM reduced blood clearance and aortic recruitment of EPCs. Both parameters were returned to control levels by CoPP treatment without affecting EPC kinetics in normal animals. These abnormalities of EPCs in DM were paralleled by reduced serum adiponectin levels, increased numbers of CECs, reduced endothelial expression of phosphorylated eNOS, and reduced levels of TM, CD31, and phosphorylated AMP-activated protein kinase (pAMPK). CoPP treatment restored all of these parameters to normal levels.

**Conclusion:**

Type II DM and its related oxidant damage hamper the interaction between the vascular wall and normal EPCs by mechanisms that are, at least partially, reversed by the induction of *HO-1* gene expression, adiponectin, and pAMPK levels.

## INTRODUCTION

Vascular disease is the principal cause of death and disability in patients with diabetes mellitus (DM). The accelerated progression of diabetic atherosclerosis is attributed to the imbalance between increased endothelial injuries and hampered endothelial repair processes [1]. The relevance and the mechanisms of the endothelial damage in DM are characterized by an abnormal control of vasomotor tone associated with an accelerated rate of cell apoptosis and sloughing [2, 3]. More recently, numerous studies—focused on the alterations induced by DM on the function of circulating endothelial progenitor cells (EPCs)—demonstrated that DM impairs EPC migration [4, 5], their differentiation to a mature endothelial phenotype, and their adhesive properties [6], as well as their capacity to proliferate [7, 8], to incorporate into vascular structures, and to contribute to vasculogenesis in the ischemic tissues of nondiabetic models [9].

Interestingly, both disorders—accelerated vascular damage and impaired EPC biology—reflect similar molecular alterations partially related to the overproduction of reactive oxygen species (ROS) [5, 10, 11]. Increased ROS levels are a well-documented mechanism of endothelial injury and a potent stimulus for the production of proinflammatory atherogenic cytokines; however, they also affect EPC mobilization, function, and survival either directly [12, 13] or via a reduction in endothelial nitric oxide synthase (eNOS) activity and nitric oxide (NO) bioavailability [12, 14]. Similarly, interventions able to potentiate tissue antioxidant power have been proven to provide a simultaneous benefit to vascular integrity and stem cell biology. In fact, upregulation of heme oxygenase-1 (*HO-1*) gene expression has been found to increase vascular repair processes [15, 16], to improve NO-dependent vasomotor control [17], to downregulate the production of interleukin-6 and tumor necrosis factor (TNF)-α [18, 19], and, finally, to reduce the evidence of cell sloughing, apoptosis, and death [2, 15]. On the other hand, this same intervention has been found to improve stem cell biology [20] via a reduction in ROS production by adipocytes, resulting in increased levels of serum adiponectin [18, 21, 22]. Indeed, reductions in circulating adiponectin have been consistently associated with obesity, insulin resistance, type 2 DM, and coronary artery disease [23, 24], whereas recent data have revealed that adiponectin plays a vascular protective role by preserving endothelial cell function in diabetic patients [25]. We have reported that enhanced vascular HO-1 expression increases serum adiponectin and enhances the concentration of phosphorylated phosphatidylinositol 3-kinase (pAKT) [17, 26]. Moreover, a large body of evidence has shown that adiponectin-mediated activation of AMP-activated protein kinase (AMPK) and pAKT increases the phosphorylation of a number of target molecules, resulting in increased glucose transport, fatty acid oxidation [19, 25], and phosphorylated eNOS (peNOS) [12] and an improvement in cell survival [12] and protection against oxidative stress [26–29]. Thus, increased oxidant damage may specifically hamper the capability of the diabetic vascular wall to recruit EPCs regardless of the availability and the function of these “spare parts.” Should this hypothesis be confirmed, it would provide a better understanding of the accelerated progression of atherosclerosis in diabetic patients. In addition, the identification of this effect could provide new insights into the improvement in stem cell-based treatments. However, despite these critical mechanistic and physiological implications, this hypothesis has never been directly tested.

Accordingly, the objective of this study was to examine whether DM specifically impairs the recruitment of EPCs, using radioactive labeling that allows detection of the presence of small numbers of EPCs in vascular structures and in different organs. To verify the role of oxidant damage in this process, we examined whether blood kinetics and vascular recruitment of normal EPCs were paralleled by ROS-dependent vascular damage in a rat model of streptozotocin (STZ) type 2 DM. Moreover, to define the mechanisms underlying these alterations, we tested the response of these indices in a phenotype preconditioned by the upregulation of HO-1 and endothelium protective signaling proteins by treatment with cobalt protoporphyrin (CoPP).

## MATERIALS AND METHODS

### Animal Models

All experiments were approved by the Institutional Animal Care and Use Committee of the University of Genoa and Pisa and conducted under the guidelines for the Care and Use of Laboratory Animals, published by the Office of Science and Health Reports, National Institutes of Health. In total, 24 male Sprague Dawley rats, 8–12 weeks of age, were included in the study: seven control rats, seven diabetic rats, five control rats treated with CoPP, and five diabetic rats treated with CoPP. Type 2 mild and stable diabetes with reduced β-cell mass was induced by i.p. administration of 210 mg/kg of nicotinamide dissolved in saline (Sigma-Aldrich, St. Louis, http://www.sigmaaldrich.com) 15 minutes before an i.p. injection of STZ, 60 mg/kg (Sigma), dissolved in citrate buffer (pH 4.5) immediately before use [30]. This procedure provides a model for noninsulin-dependent DM syndrome and is regarded as similar to human type 2 diabetes in that it has a significant insulin responsiveness to glucose and preserved sensitivity to tolbutamide due to partial pancreatic protection by nicotinamide [30]. CoPP (0.5 mg/100 g body weight) was administered s.c. once a week for 3 weeks after STZ.

### EPC Harvesting, Expansion, and Labeling

Aortas from 8-week-old Sprague Dawley rats were explanted and treated as follows for the isolation of EPCs. Tissues pieces were washed in phosphate-buffered saline (PBS), mechanically minced, and enzymatically digested after incubation for 10 minutes at 37°C in digestion solution constituted by 1× trypsin. After filtration and removal of undigested tissue, cells were washed in PBS and centrifuged for 10 minutes at 250g. Cells were then resuspended in endothelial medium (EGM2) and plated in collagen-coated six-well plates at a density of 5 × 10^6^ cells/well. Fresh medium was added 48 hours later and was replaced twice a week. In the present study, we used cells cultured for up to four passages. The lack of hematopoietic contaminants was tested via fluorescence-activated cell sorting and was negative for CD45. The endothelial nature of these cells was confirmed by the homogeneous expression of CD31, CD144, and von Willebrand factor (vWF), thus excluding contamination of mural or muscle-derived cells (data not shown).

Labeling was performed by incubating 2 × 10^6^ cultured cells with technetium (Tc)-99m-exametazime (HMPAO, Ceretec; GE Healthcare, Milwaukee, WI, http://www.gehealthcare.com) according to a procedure described in the literature [31] and modified and validated in our laboratory. Briefly, EPCs in suspension were incubated for 30 minutes at 37°C in a solution containing 3.7 MBq of Tc-99m-HMPAO. After two to three PBS washes, the cells were centrifuged and resuspended in 1 ml of saline and subdivided into three aliquots after removal of the supernatant: 2 × 450 μl for injection into two different animals and 100 μl for the estimation of the injected dose. Labeling yield, defined as cell count/(cell count + supernatant count), was in the range of 39%–43% (41% ± 1%). Labeling stability was estimated in a total of eight experiments in which cells were reattached and maintained at 37°C for 24 hours in an atmosphere containing 5% CO_2_. EPCs were then resuspended and centrifuged before counting. The decay-corrected radioactivity at 24 hours was in the range of 93%–96% of that documented soon after labeling. Similarly, the trypan blue exclusion test was used to determine preserved viability in all cultures.

### Experimental Protocol

After treatment with saline or STZ, all animals were housed for 2 months and given food and water ad libitum. Blood samples were collected weekly from the tail vein following a 4-hour fasting period in order to measure glucose levels by the glucose oxidase method (Glucocard GT-1610; Menarini Diagnostic, Firenze, Italia, http://www.menarini.com). The animals were then anesthetized with pentobarbital sodium (40 mg/kg) and a jugular vein was exposed for cannulation. Thereafter, labeled EPCs (10^6^, with a mean activity of 18.5 kBq) were infused via a femoral vein. Blood samples (100 μl) were obtained from the jugular vein catheter at 15, 30, 45, 60, 75, 90,120, 150, 180, and 240 minutes after cell injection and stored in preweighed vials containing 5 μl of heparin. In all cases, the catheter was washed with a corresponding volume of heparinated saline. All procedures were performed using Hamilton syringes (LaboIndustria SpA, Padoa, Italy, http://www.laboindustria.com). At the end of the study period, all animals were sacrificed and the following organs were harvested: lungs, spleen, liver, heart, and aorta. All blood samples were weighed with a precision balance and centrifuged for 5 minutes at 3,000 rpm to remove serum. Radioactivity of the centrifuged samples was measured in a gamma counter and results are expressed as a percentage of dose per ml of blood, considering a blood density of 1.05 g/ml. Blood clearance of labeled injected EPCs was evaluated by the hourly decrease in radioactivity concentration in the blood. Organ radioactivity was expressed as a percentage of the injected dose.

### Analysis of Blood Variables

Before labeled cell injection, 2-ml blood samples were obtained for subsequent analysis. Serum adiponectin (high molecular weight) was determined using an enzyme-linked immunosorbent assay (ELISA) (Pierce Biotechnology, Inc., Woburn, MA, Rockford, IL, http://www.piercenet.com). Insulin levels were determined by an ELISA method (Mercodia, Uppsala, Sweden, http://www.mercodia.com). Detection and quantification of circulating endothelial cells (CECs) were performed using monodispersed magnetizable particles (Dynabeads CELLection Pan Mouse IgG kit) obtained from Invitrogen (Carlsbad, CA, http://www.invitrogen.com). Typically, 100 μl of bead suspension was noncovalently coated with 10 μg/ml of RECA-1 (Novus Biologicals, Littleton, CO, http://www.novusbio.com), a panrat endothelial cell-specific monoclonal antibody, and CECs were determined as previously described [2, 32]. Finally, the concentration of oxidized proteins was assayed using ELISA kits (Cayman Chemical Co, Ann Arbor, MI, http://www.caymanchem.com).

### Western Blot Analysis of Aortas for HO, eNOS, AMPK, pAMPK, AKT, and pAKT

Frozen aortas were pulverized under liquid nitrogen and placed in a homogenization buffer (10 mmol/l phosphate buffer, 250 mmol/l sucrose, 1 mmol/l EDTA, 0.1 mmol/l phenylmethylsulfonyl fluoride (PMSF), and 0.1% vol/voltergitol, pH 7.5). Homogenates were centrifuged at 27,000g for 10 minutes at 4°C, the supernatant was isolated, and protein levels were visualized by immunoblotting with antibodies against HO-1 and HO-2 (Stressgen Biotechnologies Corp., Victoria, BC, Canada, http://www.stressgen.com). Antibodies against AKT, AMPK, pAMPK, and pAKT were obtained from Cell Signaling Technology, Inc. (Beverly, MA, http://www.cellsignal.com), eNOS was from Santa Cruz Biotechnology Inc. (Santa Cruz, CA, http://www.scbt.com), and 3-NT was from Upstate cell signaling solutions (Chicago, IL, http://www.upstate.com). Antibodies were prepared at the following dilutions: HO-1 and HO-2, 1:1,000; 3-NT, 1:1,000; eNOS, pAMPK, and pAKT, 1:5,000 and 1:1,000 for the cellular apoptotic mediators.

Briefly, 20 μg of aortic tissue lysate supernatant was separated by 12% SDS-PAGE and transferred to a nitrocellulose membrane. Immunoblotting was performed as previously described [17]. Chemiluminescence detection was performed with the Amersham ECL detection kit (Amersham, Piscataway, NJ, http://www.amersham.com), according to the manufacturer's instructions.

### Immunohistochemical Staining

Thrombomodulin (TM) and CD31 were determined immunohistochemically as previously described (TM [33], CD31 [16]).

### Determination of HO Activity

Frozen aortas were pulverized under liquid nitrogen and placed in homogenization buffer (10 mmol/l phosphate buffer, 250 mmol/l sucrose, 1 mmol/l EDTA, 0.1 mmol/l PMSF, and 0.1% vol/voltergitol, pH 7.5). Homogenates were centrifuged at 27,000g for 10 minutes at 4°C. HO activity was determined and expressed as bilirubin formation per mg protein/hour, as previously described [26] using an Extinction Coefficient of 40 mM^−1^cm^−1^.

### Statistical Analyses

Data are presented as the mean ± standard error for the number of experiments. Statistical significance (*p* < .05) between experimental groups was determined by the Fisher method of analysis of multiple comparisons. For comparison between treatment groups, the null hypothesis was tested by a single-factor analysis of variance for multiple groups or an unpaired *t*-test for two groups.

## RESULTS

### Effect of CoPP on Body Weight, Plasma Glucose, and Plasma Insulin

Animals treated with STZ exhibited diabetes that was confirmed by significantly (*p* < .05) elevated levels of plasma glucose compared with control animals. Plasma insulin and body weight were unaffected by STZ treatment. CoPP treatment had no effect on these variables in both diabetic and nondiabetic animals (Table 1).

**Tabel 1:** General features of the experimental models

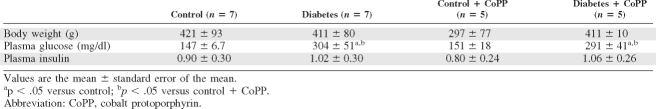

### Kinetics and Homing of EPCs

**Figure 1:**
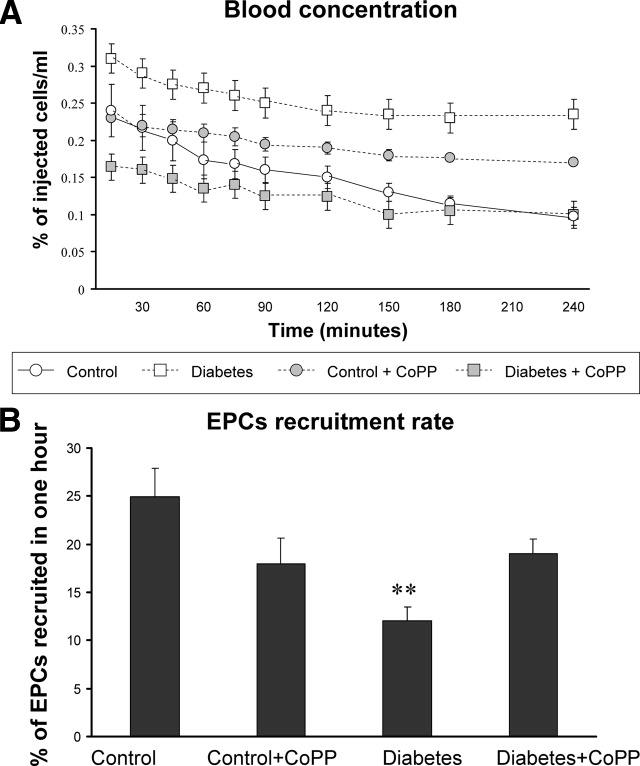
EPC kinetics in blood. **(A)**: Time concentration curves of radioactivity in the blood are shown expressed as a percentage of the dose (and thus, of the labeled injected EPCs). Diabetes was associated with a prolonged persistence of EPCs in the blood, as seen by the higher values of radioactivity (and thus, of EPC concentration) at each time point. Induction of heme oxygenase-1 (*HO-1*) gene expression by CoPP treatment (gray symbols) selectively accelerated EPC clearance from the blood in diabetic animals (squares), without an effect on nondiabetic rats (circles). **(B):** Whole body clearance of EPCs. Diabetes was associated with a marked reduction in EPC recruitment in the body, expressed by a prolonged persistence of these cells in the blood (**p < .01), whereas CoPP treatment restored this variable to normal values. Abbreviations: CoPP, cobalt protoporphyrin; EPC, endothelial progenitor cell.

**Figure 2:**
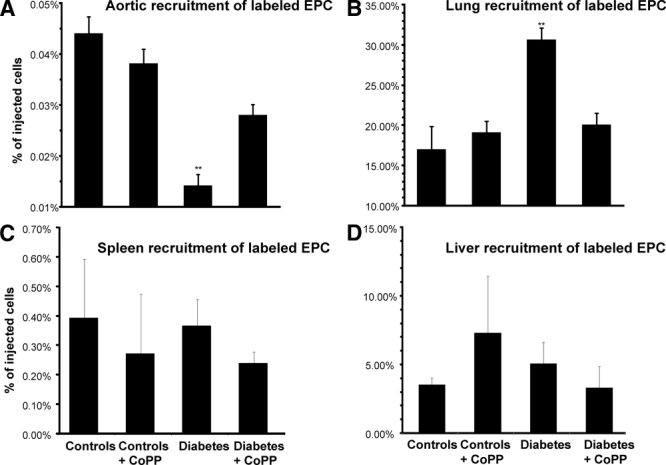
EPC recruitment rate in the aorta, lung, spleen, and liver. The recruitment rate was unaffected by diabetes and CoPP treatment in the liver and spleen. Reciprocal results are seen in the aorta and lung of diabetic animals. **p < .001, diabetic rats versus controls. Abbreviations: CoPP, cobalt protoporphyrin; EPC, endothelial progenitor cell.

As displayed in Figure 1A, the persistence of EPCs in blood was longer in diabetic than in control rats, as seen by a higher concentration of EPCs in the blood of control compared with diabetic animals (*p* < .01) throughout the study period. CoPP treatment reduced the blood concentration of labeled EPCs (*p* < .01) in diabetic animals to values close to those in control animals, while producing no significant effect on normal rats. The disappearance rate of EPCs from blood confirmed this concept: the blood concentration of injected EPCs decreased every hour by 8% ± 1% in diabetic animals and by 25% ± 2% in control animals (*p* < .01). Treatment with CoPP increased the blood clearance of EPCs in diabetic animals (to 20% ± 2%; *p* < .01 versus diabetes without CoPP, not significant versus control) but not in naive animals (to 19% ± 1%, not significant versus control) over the first hour, and it remained greater than in diabetic animals throughout the period of study (Fig. 1B). The abnormally low blood clearance of EPCs in diabetic animals was paralleled by a reduction in vascular recruitment of these cells, as shown by lower aortic uptake in diabetic versus control animals (0.01% ± 0.001% versus 0.04% ± 0.001% of injected cells, respectively; *p* < .01) (Fig. 2A). CoPP treatment significantly increased EPC recruitment in diabetic rats (to 0.03% ± 0.001%; *p* < .01 versus nontreated diabetic rats) but not in naive rats (0.03% ± 0.0005%, not significant versus control) (Fig. 2A). In contrast, the effect of long-lasting DM on lung sequestration of injected EPCs was the opposite: it was larger in diabetic than in control animals (31% ± 1.5% versus 17% ± 1.4%, respectively; *p* < .05). Again, CoPP treatment returned lung uptake of EPCs to normal levels in diabetic animals (to 20% ± 1.5%; *p* < .05 versus nontreated diabetic animals) but had no effect in naive rats (19% ± 1.5%%, not significant versus controls) (Fig. 2B). Finally, neither diabetes nor CoPP treatment affected EPC uptake in the spleen or liver (Fig. 2C, D).

### Effect of CoPP on Serum Oxidative Stress

**Figure 3:**
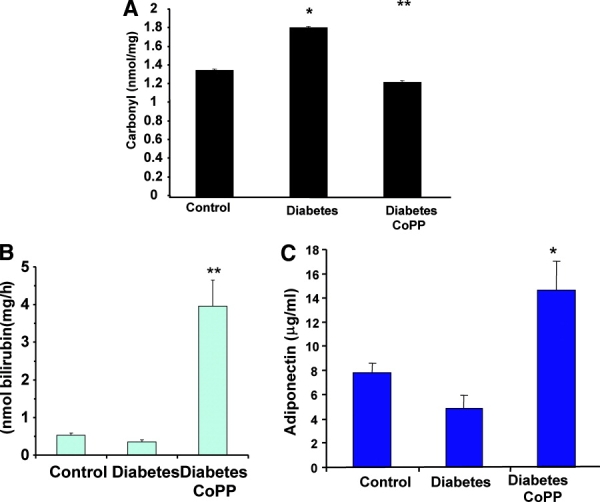
Molecular effects of CoPP treatment. **(A):** Enzyme-linked immunosorbent assay for the presence of serum oxidized proteins. CoPP restored normal values of oxidized proteins in the serum of diabetic rats; *n* = 5, *p < .01 versus control, **p < .01 versus diabetes. **(B):** Heme-oxygenase activity in the aorta, *n* = 4. CoPP treatment markedly increased Hemeoxygenase activity in diabetic rats, **p < .001 versus control and diabetic animals. **(C):** Adiponectin levels in the plasma of control rats, diabetic rats, and diabetic rats treated with CoPP. CoPP markedly increased serum concentration of this hormone in diabetes: *n* = 4. *p < .05 control versus Diabetic and control rats.

Oxidized protein (carbonyl) content was elevated in diabetic rats (1.75 ± 0.42 nmol/mg) compared with controls (1.10 ± 0.27 nmol/mg; *p* < .05) (Fig. 3A). CoPP treatments decreased serum oxidative stress, as seen by a decrease in carbonyl content (1.28 ± 0.27 nmol/mg).

### Effect of CoPP on HO Activity, Serum Adiponectin, and Endothelial TM and CD31 Levels

**Figure 4:**
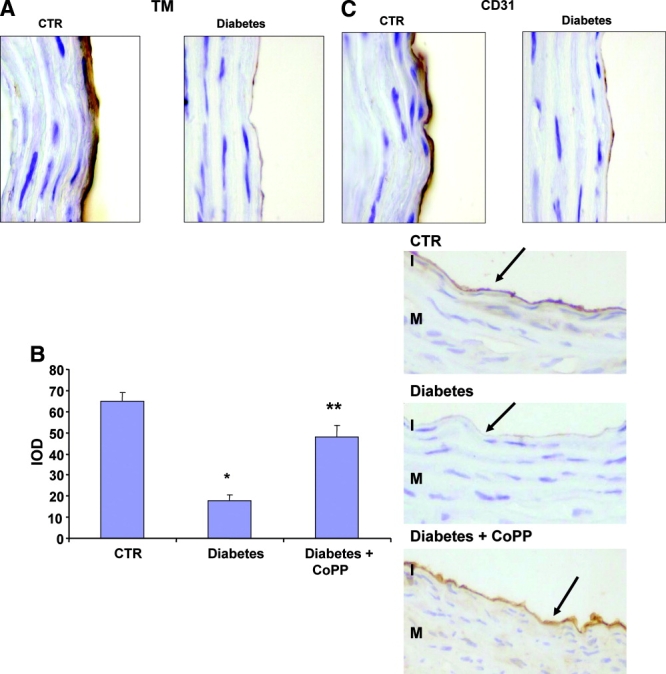
Immunohistochemical staining of TM **(A)** and CD31 **(B)** from control and diabetic animals. **(C):** Optical density analysis of TM staining in diabetic rats and its restoration by CoPP. *p < .05, control versus diabetic animals, mean ± standard error, *n* = 40. Abbreviations: CoPP, cobalt protoporphyrin; CTR, control; I, tunica intima; IOD, integrated optical density; M, tunica media; TM, thrombomodulin.

As seen in Figure 3B, HO activity was reduced in diabetic rat aortas and was increased by CoPP administration in aortas isolated from diabetic rats (from 0.81 to 3.58 nmol/60 minutes per mg protein) (Fig. 3B). This was associated with an increase in HO-1 protein levels (results not shown). Similarly, diabetic animals had decreased serum levels of adiponectin compared with control animals. Again, CoPP treatment significantly increased serum adiponectin levels in diabetic animals to levels twofold higher than those found in control animals (Fig. 3C). Immunohistochemical staining for TM was localized within the endothelial cell cytoplasm. TM staining was strong in the intima of control rats whereas diabetic rats demonstrated moderate to weak staining (Fig. 4 A). CoPP treatment restored TM expression in diabetic rats to the level of staining seen in aortas from control animals (Fig. 4A). Optical density analysis of immunohistochemical staining provided quantification of the changes in TM expression. Similar results were seen for CD31 (Fig. 4C, 4D).

### Effect of CoPP on CEC Fragmentation

**Figure 5:**
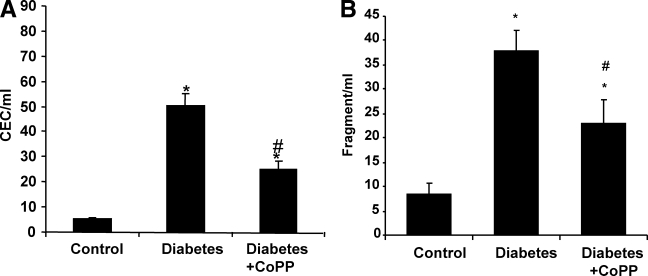
Reduction of endothelial damage by CoPP treatment. **(A):** The number of CECs increased significantly in STZ-induced diabetic rats (*p* < .05) relative to control rats. Administration of the heme oxygenase-1 inducer CoPP decreased the number of CECs, *n* = 6. *p < .05 versus controls; #*p* < .05 versus diabetic rats. **(B):** Similarly, endothelial cell membrane fragments in blood obtained from control rats were increased in diabetic rats and decreased after CoPP treatment, *n* = 5. *p < .001 versus controls; #*p* < .001 versus diabetic rats. Abbreviations: CEC, circulating endothelial cell; CoPP, cobalt protoporphyrin; STZ, streptozotocin.

Using immunomagnetic isolation, we identified a well-defined population of cells in the peripheral blood of normal and diabetic rats, which reproducibly stained positive for vWF, CD34, and Ulex europaeus agglutinin-1. These CECs were larger than other blood cells (10–50 μm in length) and had an oval or round shape (results not shown). Immunofluorescence staining was computed as the integrated optical density and was measured in four to seven samples for each experimental group. Figure 5A shows that DM was associated with a marked increase in the number of CECs (48 ± 12 cells/ml versus 7 ± 4 cells/ml, respectively; *p* < .01). CoPP treatment significantly decreased the number of CECs (23 ± 5 cells/ml; *p* < .01 compared with nontreated diabetic rats). Figure 5B displays the number of endothelial cell membrane fragments, an indicator of cell apoptosis. There was an 8- to 12-fold increase in cell fragmentation in diabetic rats compared with controls (*p* < .001). *HO-1* upregulation by CoPP diminished the number of fragments (*p* < .001) compared with nontreated diabetic animals.

### Effect of CoPP on eNOS and pAMPK

**Figure 6:**
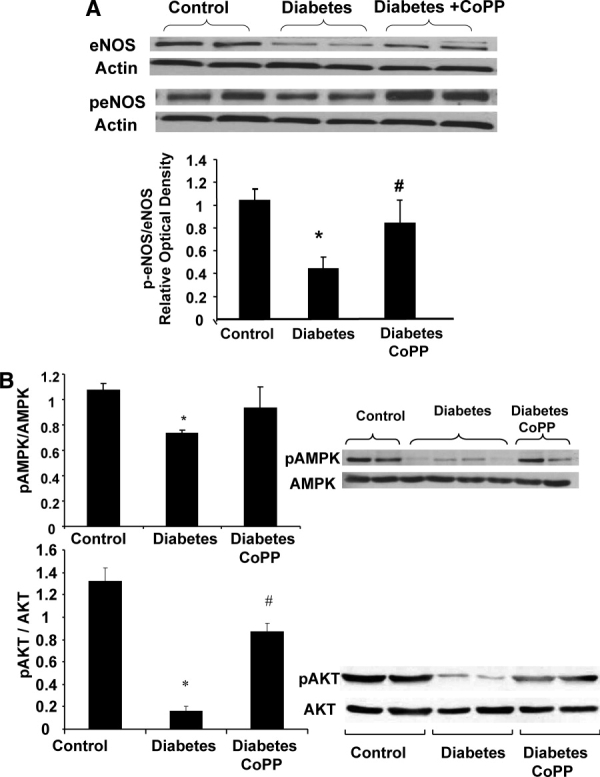
Effect of CoPP treatment on endothelial gene expression. **(A):** Western blot and densitometry analysis of eNOS and peNOS from control, diabetic, and CoPP-treated diabetic rat aortas. Quantitative densitometry evaluation of eNOS and peNOS in the aorta was determined. Each bar represents the mean ± standard error of four experiments. *p < .001 for diabetic versus CoPP-treated diabetic rats. **(B):** Effect of diabetes and *HO-1* expression on pAKT and total AKT in the aorta's proteins, and AMPK and pAMPK. Quantitative densitometry evaluations in aorta tissue homogenates of pAKT and pAMPK are expressed as a ratio to HO-2. *p < .01, diabetic rats versus control rats or CoPP-treated diabetic rats. Abbreviations: AKT, phosphatidylinositol 3-kinase; AMPK, AMP-activated protein kinase; CoPP, cobalt protoporphyrin; eNOS, endothelial nitric oxide synthase; HO, heme oxygenase; p, phosphorylated.

Finally, we verified if the restoration of TM and CD31 in the aortas of diabetic rats was associated with increases in eNOS protein levels. As seen in the Western blot analysis and densitometry evaluation (Fig. 6A), CoPP administration to diabetic rats resulted in a significant increase in eNOS protein (*p* < .05) compared with untreated diabetic rats. Thus, the ability of HO-1 expression to augment vascular repair in vivo may be due, in part, to an increase in the levels of eNOS.

As expected, induction of diabetes resulted in no significant changes in AKT but significant decreases in both pAKT and pAMPK. Upregulation of *HO-1* by CoPP administration increased the levels of both the pAKT and pAMPK proteins. As seen on densitometry analysis, pAKT was significantly increased in diabetic rats treated with CoPP, compared with nontreated diabetic rats (*p* < .05). Similarly, a significant increase in pAMPK was seen in diabetic rats treated with CoPP (*p* < .01) (Fig. 6B) when compared with nontreated diabetic animals.

## DISCUSSION

This study is the first in vivo characterization of the effects of DM on the trafficking of normal EPCs. The data show that DM markedly impairs the vascular recruitment of normal EPCs, prolongs their residence in circulating blood, reduces their aortic recruitment, and increases their lung sequestration. All these abnormalities are corrected by induction of the *HO-1* gene, which, in contrast, does not affect EPC kinetics and distribution in normal animals. In addition, the increased EPC recruitment due to the antioxidant effect of *HO-1* gene induction results in the reprogramming of EPC signaling components, as manifested by the restoration of the activities of pAMPK and peNOS and an increase in vascular repair in diabetic animals. These data extend our knowledge on the interaction(s) between stem cells and tissues under disease conditions and show that an elevated ROS concentration specifically impairs signaling by the diabetic vascular tree that is necessary to recruit even normal EPCs.

In the present study, we directly labeled EPCs with Tc-99m-HMPAO using a method clinically validated to obtain radioactive leukocytes and adapted to obtain stable binding throughout the experiment without any significant effect on EPC viability and function [34]. According to tracer kinetic theory, monitoring radioactivity in blood cells and measuring its final uptake in each organ estimates the number of cells cleared from the blood per unit time and the number of cells recruited by each organ. The loss of about 64% of the cells from the circulation in 4 hours (Fig. 1) indicates that the recruitment of EPCs is an unexpectedly high rate process in normal animals and that the time span of our study is large enough to detect a significant impairment induced by DM. Similarly, the rapid nature of this phenomenon accounts for the number of injected cells that resulted in a concentration of EPCs in the first blood sample well within the physiological range (0.16%–0.32% of the dose/ml, or 16–32 EPCs/μl) documented in clinical studies [34].

The decreased aortic recruitment contrasts with the increased uptake of EPCs in diabetic lungs. Despite its paradoxical appearance, this difference is better understood when the uptake mechanisms in the two structures are considered. The aorta is exposed to laminar flow and no physical sequestration of cells can occur. Accordingly, radioactivity (cell) uptake has to be interpreted as the result of cell adhesion. In contrast, every substance injected i.v. has to cross the pulmonary capillaries to be available for distribution in the body and to be present in circulating blood. Therefore, injection of particulate tracers can result in pulmonary sequestration of those particles—cells—that physically cannot cross tubes of 7 μm in diameter. This concept represents the basis of lung perfusion scintigraphy and has been confirmed for i.v. injection of labeled mesenchymal cells [31]. Accordingly, the increased sequestration of EPCs in diabetic lungs is explained by the pulmonary microvascular alterations reported in STZ-treated rats characterized by a high prevalence of capillary collapse associated with focal thickening of basal lamina in the capillary endothelium [35].

Regarding the aims of the present study, the large number of cells sequestered in diabetic lungs has two important implications. First, it prevents the evaluation of EPC adhesion in the pulmonary endothelium due to the large contamination of wedged radioactive cells. Second, lung removal of labeled EPCs from the circulation results in reduced numbers of circulating EPCs in diabetic rats. Accordingly, the elevated concentration of radioactivity in these animals indicates that the recruitment of EPCs throughout the diabetic vascular system is even more impaired than expected. This is confirmed by the effect of CoPP, which selectively reversed EPC recruitment in the lung and aorta of diabetic animals. Obviously, the kinetic characterization of EPCs collected from diabetic animals and injected into nondiabetic animals may provide insights into vessel–EPC signaling. However, the expansion of EPCs in vitro may reduce the effect of the “diabetic memory” of injected cells, thus hampering the interpretation of the data.

Together with the prolongation of EPC persistence in the blood in DM and its response to CoPP, the reduced radioactivity uptake in the aorta indicates that, besides damaging the mature endothelium, elevated ROS concentrations also impair the recruitment of EPCs by diabetic vessels. Therefore, these data extend previous knowledge of the relationship between downregulation of *HO-1* activity and accelerated progression of atherosclerotic lesions in native vessels [36]. Similarly, they provide new insights into the mechanism underlying the beneficial effect of HO-1-derived CO and bilirubin on endothelial cell death and apoptosis both in vitro and in vivo [3, 19]. In fact, the salutary effect of CoPP was confirmed by increased endothelial levels of CD31 positivity and TM, whose downregulation is an important index of endothelial cell death and progression of atherosclerosis [13, 37]. In agreement with this concept, the increased expression of CD31 and TM was associated with a reduction in the number of CECs and cell fragments in CoPP-treated diabetic animals, confirming the protective role of *HO-1* induction and adiponectin on EPC recruitment by the diabetic endothelium.

Endothelial cell dysfunction, demonstrated by the reduced expression of CD31 [38, 39] and/or TM [37, 38], has been reported within atherosclerotic blood vessels. A *CD31* gene abnormality has also been implicated in the pathogenesis of both atherosclerosis and myocardial infarction (MI) [40]. Furthermore, a reduction in plasma TM has also been associated with an increased risk for MI [13]. Conversely, increased expression of TM has been shown to limit thrombus formation as well as neointimal growth [41]. The increase in TM and CD31 positivity limits neointimal formation and endothelial cell dysfunction [38]. The diminished function of vascular endothelial cells that occurs with diabetes [38, 41] is accompanied by a reduction in EPC function [16, 42], which further impacts the integrity of the intact endothelial lining. The increases in pAMPK and pAKT induced by the chronic administration of CoPP are likely major factors in EPC protection and an indication of the restoration of vascular integrity. This is of particular interest because reversal drugs, such as the statins, known for their antiatherosclerotic properties, have been shown to increase both HO-1 and eNOS.

The restoration of normal rates of EPC recruitment after CoPP treatment was paralleled by increased levels of endothelial pAMPK, eNOS, and peNOS. This latter observation seems of particular relevance due to the pivotal role of reduced NO bioavailability in the altered regulation of vasomotor tone, cell adhesion, angiogenesis, and vasculogenesis that characterizes the tissues of diabetic patients [43, 44]. This is further amplified by the specific alteration in eNOS expression documented in the bone marrow microenvironment, leading to reduced mobilization of EPCs in DM [45].

The present data do not elucidate whether the reduced expression of eNOS is the cause or the effect of the decreased vascular recruitment of EPCs in DM. However, the relevance of vessel stem cell interactions and their alterations provide a new clue to understanding the effect of drugs, such as statins, known for their antiatherosclerotic properties, and their ability to increase both HO-1 [46] and eNOS [2, 47]. Similarly, the present data do not permit us to conclusively identify the molecular mechanism underlying the increased eNOS expression resulting from upregulation of *HO-1*. However, at least two points must be considered: first, CoPP treatment did not significantly decrease the severity of hyperglycemia in our animal model and did not affect serum insulin levels; second, in contrast, circulating adiponectin levels were increased. This finding confirms previous observations [18, 19] and seems of particular relevance as adiponectin has been reported to possess a vascular protective role [25], to preserve endothelial cell function in diabetic subjects, to increase eNOS activity, and to reduce the expression of eNOS inhibitors such as TNF-α [48]. The association of increased serum adiponectin levels and increased EPC recruitment closely fits with previous findings by Ouchi et al. [49], who reported a critical role for adiponectin in endothelial cell survival and function via the activation of eNOS, pAKT, and pAMPK. These enzymes enhance peNOS levels [12, 50, 51], suggesting that EPC recruitment is a result of the reprogramming of specific metabolic pathways and their signaling components, such as HO-1, pAMPK, and peNOS, in a manner that enhances vascular repair.

## CONCLUSIONS

The present data confirm the mechanisms underlying the endothelial damage characteristic of DM and suggest that they also are responsible for the impaired capability of the vascular wall to recruit EPCs. The abnormal expression of eNOS, TM, and CD31 was associated with increased numbers of both CECs and cell fragmentation, confirming the presence of structural endothelial damage. Despite this evidence, we found that the capability to recruit EPCs was reduced in diabetic vessels. Moreover, the beneficial effect of *HO-1* gene induction on the endothelial phenotype was selectively observed in diabetic rats. Accordingly the selective acceleration of EPC recruitment observed in these animals cannot be ascribed to nonspecific vascular damage induced by CoPP treatment and upregulation of *HO-1* and the prevention of radical generation. Rather, it strongly indicates that HO-1-preconditioned endothelium is able to recruit more normal EPCs via an increase in adiponectin and pAMPK. Accordingly, these results indicate that the signaling disorder that interferes with the ability of the vascular tree to recruit EPCs represents an important mechanism that underlies the accelerated progression of atherosclerosis in DM. In addition, it has the potential to amplify endothelial damage and to reduce the number and the function of circulating EPCs observed in these patients [52]. A deeper understanding of the mechanisms involved will provide new approaches for the treatment of diabetic atherosclerosis and improve the effectiveness of cell-based treatment of vascular diseases.

## ACKNOWLEDGMENTS

This work was supported by the National Institutes of Health grants HL55601, DK068134, and HL34300 (N.G.A.); by the CNR Medical Department and Cardiopulmonary project, the Scuola Sant'Anna (A.L.'A.); by a grant from the University of Genoa (progetto d'Ateneo 2006, G.S.); and by a grant from the Compagnia di San Paolo Torino (F.F.), Progetto CARIGE Cellule Staminali (F.F.), Progetto CARIGE (Microcircolo e diabete), Regione Liguria (Limonte Project), and the Ministero della Salute (Ricerca Finalizzata Ministeriale 2005). Finally, the authors are grateful to Prof. Umberto Marinari and Prof. Maria Adelaide Pronzato for their kind help in conducting some of the experiments described in this manuscript.

## DISCLOSURE OF POTENTIAL CONFLICTS OF INTEREST

The authors indicate no potential conflicts of interest.
